# Radiolabelling of Antigen and Liposomes for Vaccine Biodistribution Studies

**DOI:** 10.3390/pharmaceutics2020091

**Published:** 2010-03-31

**Authors:** Malou Henriksen-Lacey, Vincent Bramwell, Yvonne Perrie

**Affiliations:** School of Life and Health Sciences, Aston University, Aston Triangle, Birmingham, B4 7ET, UK

**Keywords:** liposomes, vaccines, radiolabelling, pharmacokinetics, biodistribution

## Abstract

A relatively simple and effective method to follow the movement of pharmaceutical preparations such as vaccines in biodistribution studies is to radiolabel the components. Whilst single radiolabelling is common practice, in vaccine systems containing adjuvants the ability to follow both the adjuvant and the antigen is favourable. To this end, we have devised a dual-radiolabelling method whereby the adjuvant (liposomes) is labelled with ^3^H and the antigen (a subunit protein) with ^125^I. This model is stable and reproducible; we have shown release of the radiolabels to be negligible over periods of up to 1 week in foetal calf serum at 37 °C. In this paper we describe the techniques which enable the radiolabelling of various components, assessing stability and processing of samples which all for their application in biodistribution studies. Furthermore we provide examples derived from our studies using this model in tuberculosis vaccine biodistribution studies.

## 1. Introduction

### 1.1. The origins and applications of radioisotopes

The history of using radioisotopes as tracer molecules dates back to 1913 when George De Hevesy was struggling to separate a mixture of lead and Radium D. Unbeknown to him, this was an impossible feat as Radium D is actually a lead isotope (Pb_210_) and therefore inseparable. However, it was his struggles with this situation with led (no pun intended) to the first use of radioisotopes as tracer molecules [1]. In 1935 De Hevesy published his works showing that radioisotopes could be used as tracer molecules in biological systems [2]. Nowadays public exposure to radioisotopes includes both therapeutic and diagnostic uses, for example barium meals and for treatment of cancers. In the laboratory setting radioisotopes are generally used as tracer molecules in both *in vitro* and *in vivo* experimentation. With regards to their pharmaceutical application, radioisotopes are particularly useful for studying the pharmacokinetics and/or biodistribution of substances. By associating a small ‘tracer’ amount of radioisotope at a known concentration to drugs or vaccines, the presence and quantity of the initial drug or vaccine can be determined.

### 1.2. Radiolabelling in the lab

A common problem encountered by researchers in this field is the ability to acquire pre-labelled substances, and whilst many life science and technology companies produce generic radiolabeled compounds such as albumins, amino acids and cholesterol, the ability to self-label the molecule of choice is appealing as it allows the user to have more control over aspects such as quantity required, concentration of both the drug/vaccine and the radioisotope, and the date required (as radioisotope decay and/or breakdown from associated molecules is often a problem).

When choosing to radiolabel a substance, the type of radioactive emission, energy emitted and half-life of the radioisotope must all be considered. Table 1 outlines some radioisotopes frequently used in biosciences and their respective properties. The most commonly used radioisotopes are ^3^H and ^14^C primarily due to their abundance, facility to conjugate to biological molecules and extensively documented use. Both are beta (β) particle emitters with moderate energy ranges and considerably long half lives. However as both radioisotopes emit β-particles and their energy ranges significantly overlap, their combined use in the same application is often difficult as detection instruments must separate one radioisotope from the other. To overcome this problem, a combination of β- and γ-emitters can be used, or the use of two β-emitters but with substantially different energy ranges, such as ^3^H and ^32^P. A further advantage of combining ^32^P with other radioisotopes in dual-radiolabelling studies is that ^32^P emits β-particles at such a high energy level that a scintillation cocktail is not required (for all other β-particle emitters a scintillant is required to transfer energy emitted from the radioisotope to the detector). Known as Cherenkov counting, this method shares a similarity with radioactive detection from γ-emitters in that their activity can be detected directly in the sample without the need of chemical or heat degradation to produce clear and colourless samples.

**Table 1:** Common laboratory radioisotopes and their uses.

*Radioisotope*	*Decay emission*	*Half-life*	*Application*
**^3^H**	β	12.3 years	Amino acid labelling, cell proliferation assays
**^14^C**	β	5730 years	Amino acid labelling
**^35^S**	β	87.4 days	Nucleotide, amino acid labelling
**^51^Cr**	γ	27.7 days	Cytotoxicity assays
**^32^P**	β	14.3 days	Nucleotide labelling
**^33^P**	β	25.4 days	Nucleotide labelling
**^90^Y**	β, γ	2.67 days	Therapeutic
**^125^I**	γ	60.1 days	Protein labelling

### 1.3. Application of radioisotopes in vaccine studies

Vaccine development in recent years has focused on the use of subunit proteins as opposed to killed or attenuated microorganisms, predominantly due to safety. However, whilst these vaccines display improved safety profiles, often their ability to successfully immunise the host is reduced. Adjuvants are therefore increasingly common additions to subunit vaccines and whilst they improve the immunogenicity of the subunit protein, the method in which they work is varied and still not fully understood making their classification somewhat problematic [3]. Like in many other sub-domains of pharmaceutical research, the end goal would be the development of a system of rules that could limit the need to extensively screen drug candidates and combinations. With regards to adjuvants, due to their varied results with different antigens it is not possible to have one adjuvant for numerous antigens – each antigen-adjuvant combination is treated as a separate drug system. In an attempt to increase our understanding of these systems, their distribution *in* vivo can be followed. In fact, EU guidelines on adjuvants destined for human vaccines recommend completing distribution studies for a comprehensive understanding of the method of adjuvant action [4].

Work in our lab has focussed on the use of liposomes as adjuvants and transfection agents for a range of disease models [5,6,7,8]. The bilayered structure of liposomes composed of hydrophobic and hydrophilic domains provides a versatile structure; molecules such as cholesterol or immunomodulators can intercalate into the hydrophobic bilayer or be incorporated between bilayers in the hydrophilic aqueous domain. Furthermore, inclusion of charged lipids into the bilayer results in a liposome surface charge therefore allowing electrostatic interactions with proteins and DNA. This flexible bilayer structure makes liposomes ideal systems for addition of commercially available radiolabelled lipids. Through appropriate lipid choice, the addition of ‘tracer’ amounts of such radiolabelled lipids will not significantly alter the physical or chemical structure of the liposome, a property which is especially important with regards to liposomes as adjuvants. In contrast to dyes included in the internal aqueous domain which may ‘leak’ from the liposome, the use of membrane incorporated lipids is more appealing as retention of lipids within liposome bilayers is generally high until major liposome degradation occurs.

In addition to tracing the adjuvant, detection of the antigen is equally important if a greater understanding of how these two components work together is to be had. However the epitope of subunit protein vaccine antigens must be retained for the vaccine to successfully immunise the host, therefore, radiolabelling provides a preferable alternative to fluorescent labelling as unlike the addition of fluorophores to molecules, which are often bulky moieties, can have a significant impact on properties such as pI, molecular weight, solubility and protein folding, the addition of small radioisotopes generally does not alter the structure or kinetics of the molecule in question. Furthermore, fluorescent labelling is highly dependent on pH and the presence of principally primary amine functional groups [9]. This in itself can pose a problem in formulations using Tris buffer which is a primary amine and therefore cannot be used in any stages concerning fluorescence labelling. Whilst several groups describe the use of singular radiolabelled components in biodistribution and pharmacokinetic studies [10,11,12,13,14], there are few documented dual-radiolabelled studies. With regard to reducing studies on animals, dual-radiolabelling as opposed to single component radiolabelling is more favourable as the number of animals required is reduced and is therefore in keeping with the 3 R’s of refinement, reduction and replacement.

### 1.4. Practicalities of using radioisotopes

The stability of pre-radiolabelled compounds and molecules varies greatly between compounds and is an important consideration when conducting pharmacokinetic and biodistribution studies. Addition of sufficiently active but old and decomposing radiolabelled compounds will result in analogous results with ‘free label’ giving inaccurate readings. Typically gel filtration columns, centrifugation or dialysis are used to remove unbound radioisotopes after self-radiolabelling of substances. A disadvantage therefore of self-labelling compounds destined for *in vivo* studies is the reduction in sterility with each additional step.

For the purpose of demonstrating the application of radiolabelling vaccines, the methods outlined below describe liposomes composed of the cationic lipid DDA and the immunomodulatory molecule TDB. This system, termed DDA:TDB, has been extensively analysed in our lab [6,15,16,17,18] and the results shown here were used to investigate the biodistribution of this liposome system as an adjuvant in vaccine studies.

## 2. Materials and Methods

### 2.1. Materials

Dimethyldioctadecylammonium bromide (DDA) and trehalose 6,6’-dibehenate (TDB) were purchased from Avanti Polar Lipids, Inc. (Alabaster, AL). Methanol and chloroform (both HPLC grade) were purchased from Fisher Scientific (Leicestershire, UK). Tris-base, obtained from IDN Biomedical, Inc (Aurora, Ohio) was used to make Tris buffer and adjusted to pH 7.4 using HCl; unless stated otherwise Tris buffer was used at 10mM, pH 7.4. Hydrogen peroxide, ovalbumin, Sephadex™ G-75 and bicinchoninic acid protein assay (BCA) components were purchased from Sigma Aldrich (St. Louis, MO). Ag85B-ESAT-6 was a kind gift from Statens Serum Institut, Denmark. Foetal calf serum (FCS) was from Biosera, UK. Dialysis membrane (12-14 kDa pore size) was from Medicell International Ltd, UK. For radiolabelling, l-3-phosphatidyl[*N-methyl*-^3^H]choline, 1,2-dipalmitoyl (^3^H-DPPC) was obtained from GE Healthcare (Amersham, UK), Pierce iodination tubes were from Pierce Biotechnology (Rockford, IL) and ^125^I (NaI in NaOH solution), SOLVABLE™ and Ultima Gold™ scintillation fluid were purchased from Perkin Elmer (Waltham, MA).

### 2.2. Radiolabelling of liposomes

To follow the movement of liposomes *in vivo* a stable radioactive tracer component was added to the liposomes which can integrate into the bilayer. The commercially available tritiated lipid DPPC was chosen as it was paramount that the addition of the tracer lipid did not affect the physicochemical properties of DDA:TDB. For this particular compound the proportion of ^3^H to DPPC was high (3 TBq/mmol) therefore enabling extremely low levels of ^3^H-DPPC to be used. The highest ratio of ^3^H-DPPC:DDA (or other lipid) used throughout our studies was for *in vivo* experiments *via* the intramuscular (i.m) injection route which involved relatively high amounts (<200 kBq) of ^3^H-DPPC. In this instance the concentration was 500 ng ^3^H-DPPC:5 mg DDA/mL. 

Liposomes were produced using the relatively simple method known as lipid-film hydration [19]. DDA (1.25 mg) and TDB (0.25 mg) were dissolved in a mixture of chloroform and methanol (9:1 v/v) in a round bottomed flask (rbf). ^3^H-DPPC (25 ng, dissolved in a 1:1 v/v mixture of toluene:ethanol) was added to the rbf. The solvents were removed using a roto-evaporator set over a water bath at 37 °C. This process took approximately 20 min after which the resulting lipid-film was flushed with N_2_ for 5 min to ensure complete solvent removal. Nine hundred microlitres of Tris buffer (10 mM, pH 7.4) was added to the rbf to rehydrate the lipid film. The rbf was covered with Parafilm® and held in a water bath (60 °C) with frequent vortexing for the following 20 min. For experiments requiring antigen, 100 μL of antigen (dissolved in an appropriate buffer to a concentration 10-fold higher than required) was added to the cooled liposomes so that adsorption *via* electrostatic interactions may take place. For *in vivo* studies, the concentration of liposomes was increased so that an intramuscular injection (50 μL) contained 250 μg DDA, 50 μg TDB and 25 ng ^3^H-DPPC.

### 2.3. Radiolabelling of antigen

**Figure 1:**
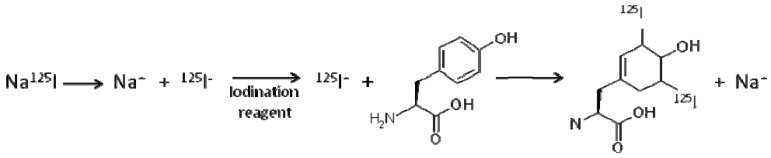
Diagram showing iodination of the amino acid tyrosine using Pierce oxidising reagent present in Pierce iodination tubes. Two possible iodination sites exist on tyrosine at the ortho ring position either side of the hydroxyl group [20].

To radiolabel protein antigen, Pierce iodination tubes containing Pierce iodination reagent (formally known as IODO-GEN tubes) were used. The tubes contain an oxidizing reagent which converts NaI into a reactive iodine molecule that can insert into the tyrosol group of tyrosine amino acids (Figure 1).

For efficient iodination the antigenic protein must therefore contain an adequate number of tyrosine amino acids, however for proteins which contain few tyrosine groups the Bolton-Hunter reagents can be used to introduce iodinatable sites. The iodination reaction is pH dependent with the optimum range being pH 6.5–8 and a 15% reduction in labelling efficiency with a pH decline of one unit [21]. It is therefore important to consider the protein and the buffer in which it is dissolved before carrying out iodination using Pierce iodination tubes. We have used the model antigen ovalbumin (OVA) which readily dissolves in Tris buffer (pH 7.4) and contains nine tyrosine groups for iodination to take place. Other examples of proteins we have used for radiolabelling are shown in Table 2.

**Table 2:** Selected proteins and their physical characteristics with relation to ^125^I-radiolabelling.

*Protein*	*Size (kDa)*	*pI*	*N^o^ Tyrosine residues*	*^125^I-labelling Buffer/pH*	*^125^I-labelling efficiency*	*% adsorption to DDA:TDB liposomes*
OVA	45	4.5	9	Tris/7.4	+++	88 %^a^
BSA	66	4.7	21	Tris/7.4	+++	N/D
Lysozyme	14.7	11	3	Tris/4	++	29 %^b, [22]^
Ag85B-ESAT-6	44	4.9	13	Tris/7.4	+++	97 %^b, [22]^

*^a^* Determined using the BCA assay; *^b^* determined using ^125^I-radiolabelling; N/D, not determined.

Iodination of proteins is a two step process: firstly protein and NaI are added to an iodination tube and left for approximately 1 h for the ^125^I to be incorporated into tyrosine groups; secondly free unbound ^125^I must be removed. For this latter process we used gel filtration chromatography using Sephadex™ G-75 beads. To make the column, 1 g of Sephadex™ G-75 was rehydrated overnight at room temperature with 20 mL Tris buffer (10 mM, pH 7.4). The bead slurry was poured into a 5 ml glass pipette; glass was used as opposed to plastic to minimise protein binding to the column and to facilitate decontamination of the pipette after use. To radiolabel the protein, the amount of protein and ^125^I required was calculated with the rule that all the protein and approximately 70 % of the ^125^I would be recovered from the column. These values were calculated in preliminary experiments whereby 1 mg OVA and 1 MBq ^125^I were radiolabelled and the unbound ^125^I was removed using a gel column (made as described above). Samples were recovered from the column in 1 minute intervals and each sample (approximately 250 μL) was measured for protein content using the BCA assay. The amount of OVA in each sample was calculated using an OVA standard curve and plotted against the amount of iodine (in counts per minute, CPM) per sample.

For *in vivo* immunisation studies, 2 μg Ag85B-ESAT-6 and approximately 100kBq ^125^I was required per dose (intramuscular injection). Therefore to make sufficient for 40 doses, for example, 80 μg Ag85B-ESAT-6 (100 μl at 800 μg/mL) and 5.71 MBq ^125^I (40 doses at 100 kBq each, plus 30 % which is counted as ‘free unbound’ ^125^I) was added to an iodination tube. This was left for 1h with intermittent swirling to ensure that the protein and ^125^I solutions were fully exposed to the iodination reagent on the tube walls. To remove the unbound ^125^I, the tap on the gel filtration column was opened and the total volume of protein-^125^I added to the top of the gel taking care to ensure that the solution flowed into the gel directly and quickly. The column was flushed with Tris buffer (10 mM, pH 7.4) and samples collected directly into γ-vials at 1 min intervals. Forty samples were collected as this represented the total column volume (~10 mL) and therefore anything obtained after this would be interacting in a non-size dependent manner with the gel medium. Each sample was counted for the presence of ^125^I using a γ-counter and Ag85B-ESAT-6 using the BCA assay. Samples positive for both ^125^I and Ag85B-ESAT-6 were pooled and the volume adjusted to 80 μg/mL Ag85B-ESAT-6 so that upon mixing with an equal volume of liposomes for i.m. vaccination (50 μL) the amount of Ag85B-ESAT-6 would be 2 μg/dose.

### 2.4. Stability studies using ^3^H-labelled liposomes or ^125^I-labelled antigen

One of the issues with radiolabelling is when quantifying their presence in samples you are actually measuring the radioisotope with the assumption it remains associated with the molecule/protein/liposome in question. Therefore it is important to ensure that the radioisotope stays associated with the molecule/protein/liposome, both ‘on the bench’ and *in vivo*. For the purpose of biodistribution studies, we devised methods to investigate the efficiency of ^3^H-DPPC retention in the liposome membrane, and the retention/release profile of ^125^I-labelled Ag85B-ESAT-6 from liposomes. Both these studies were undertaken at 37 °C in a solution of 50% foetal calf serum to simulate the *in vivo* environment.

To measure ^3^H-DPPC retention in the lipid bilayer, liposomes composed of DDA (250 μg), TDB (50 μg) and ^3^H-DPPC (25 ng) were produced as described in section 2.2 using a volume of 500 μL Tris buffer to rehydrate the lipid film. A volume of 250 μL liposomes with 250 μL of FCS/Tris buffer (50:50 v/v) were placed inside a dialysis membrane with a pore size of 12-14kDa. The membrane was sealed and placed in a 50 mL conical tube containing 40 mL FCS/Tris buffer (50:50 v/v). Dialysis was undertaken at 37 °C in a shaking water bath with 400 μL samples of the dialysing buffer being removed at various time points and being replaced with 400 μL of FCS:Tris (50:50 v/v) to maintain sink conditions. For ^3^H detection, 10 mL of Ultima Gold™ scintillation fluid was added to each 400 μl sample and counted on a scintillation counter using a basic tritium counting protocol.

^125^I-labelled protein can be used to measure protein adsorption to liposomes and the release profile from liposomes in simulated *in vivo* conditions. As opposed to dialysis we used centrifugation to measure these properties and one liposome dose (equivalent to 250 μg lipid) was prepared for each time-point of interest. Liposomes were produced without the presence of the ^3^H tracer DPPC and only rehydrated in half the normal volume so that upon addition of the ^125^I-Ag85B-ESAT-6 (20 μg/mL) the final lipid concentration would be 250 μg in 200 μL. ^125^I-Ag85B-ESAT-6 was left to adsorb to the liposomes for 1 h following which 200 μl was placed in a thick-walled eppendorf (Beckman Coulter, Polyallomer 1.5 mL tubes) with 800 μL of FCS:Tris (50:50 v/v). The eppendorf was vortexed well and either incubated at 37 °C for release studies or processed straight away to determine percentage protein adsorption. For processing, each sample was spun at 125,000 × g, 4 °C for 30 min followed by one wash using Tris buffer. The two supernatants and pellet were counted for ^125^I presence using a gamma counter. The percentage protein adsorption and/or release from liposomes were determined based on the total amount of ^125^I recovered.

### 2.5. Processing of tissues containing ^125^I and ^3^H

Biodistribution of antigens and liposomes within animal models can be traced by collecting various tissue samples and/or blood at selected time intervals. Quantification of ^125^I is relatively simple as γ-rays can pass through solid tissue and do not require a scintillant to transfer the energy emitted from the radioisotope to the detector in the machine. Detection of ^3^H is somewhat more problematic for a number of reasons. Firstly a scintillant (scintillation cocktail) is required as scintillation counters do not directly measure the radioactive particles produced but instead measure light photons which result from excitation of the scintillant. Secondly the energy emitted by radioisotopes can be absorbed by the scintillant and other chemicals added to the sample for the purposes of digestion and bleaching. This is termed chemical quench and the efficiency in detecting light photons produced by the scintillant is also reduced when colour is present in the sample (colour quenching). Finally, the counting efficiency of ^3^H is poor (typically less than 60%) so having additional factors present such as chemical and colour quench further decrease this value. Therefore, good tissue preparation is paramount and identical processing of each sample is recommended so that chemical quench is kept stable. Samples containing haemoglobin and its derivatives (e.g., blood, liver) have higher levels of colour quench than lighter tissues such as serum, muscle or brain. Incomplete tissue digestion is also common for the digestive tract due to the presence of fats or faeces containing cellulose.

The protocol described below has been used for the digestion of brain, blood, heart, lung, kidney, liver, spleen, lymph node, small intestine, muscle and dermis/hypodermis tissues. Typically samples collected were less than 100 mg in weight however for the heart, spleen and kidney the whole organ was processed and occasionally this was over 100 mg. Furthermore, as previous experiments [23] were focussed on the retention of vaccines at the site of injection (quadriceps muscle), all muscle taken from the lower leg was processed (between 200–300 mg). For the processing of blood, less than 100 mg was preferential due to the strong colour quenching. All the samples collected were fully digested and colourless using the protocol described below, the exception being that some of the blood and liver samples had a slight yellow tinge.

**Figure 2:**
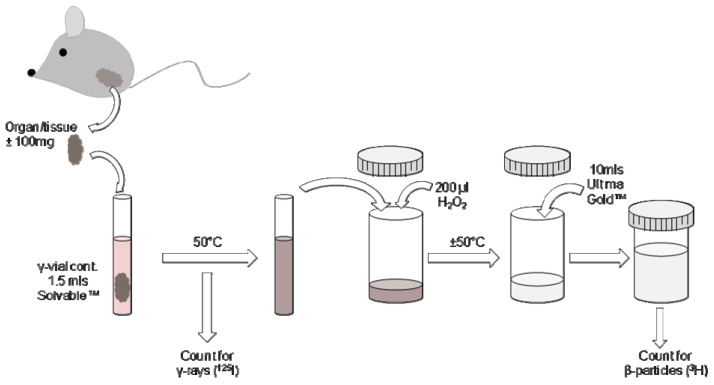
Schematic showing the processes involved in tissue processing to detect ^125^I and ^3^H presence following injection of dual-radiolabelled components.

Upon retrieval of the tissues, the material was counted directly for the presence of ^125^I-radiolabelled protein using a Cobra™ CPM Auto-Gamma® counter (Packard Instruments Company inc., IL, USA). To verify that the presence of undigested tissue and whole organs did not affect the count rate, all samples from original experiments were counted for ^125^I prior and post tissue digestion. There was no difference in the results therefore samples containing ^125^I can be counted prior or post digestion. One and a half millilitres of Solvable™ (solubilising agent) was added to each sample and a lid put on the vial. The vials were heated to 50 °C with frequent agitation of the tubes to aid Solvable™ penetration of the tissues. Once fully solubilised and cooled the tissue solutions were transferred to plastic scintillation vials; whilst glass scintillation vials may also be used, plastic vials are reported to have lower background counts and are less expensive and easier to dispose of. Two hundred microlitres of H_2_O_2_ (Sigma Aldrich) was added to each sample to bleach the solution thereby minimising colour quench. The samples were heated at 50 °C until fully decolourised and foaming had stopped. Once cooled 10 mL of Ultima Gold™ scintillation fluid was added to each vial and the samples counted using a standard ^3^H detection protocol (Figure 2).

## 3. Results and Discussion

### 3.1. Radiolabelling of proteins

**Figure 3:**
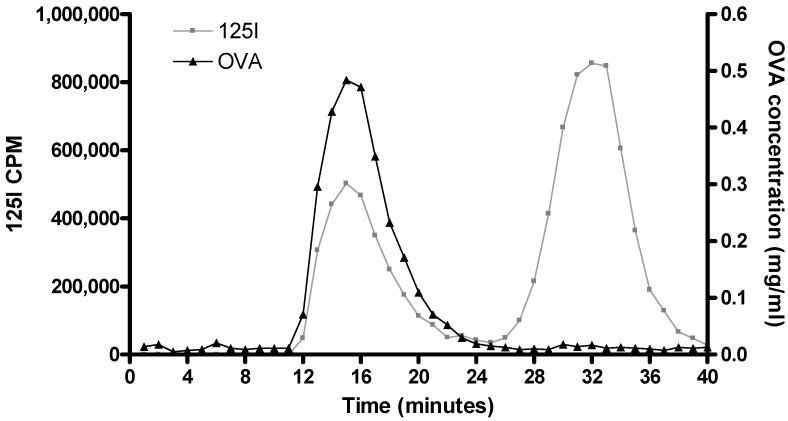
Gel filtration was used to separate unbound ^125^I from OVA antigen. The graph shows the presence of ^125^I (left y-axis, -♦-) and OVA (right y-axis, -■-) in 40 samples collected from a Sephadex™ G-75 column measuring approximately 8 cm.

As previously mentioned, one of the main benefits of radiolabelling is its non-intrusive nature whereby the original molecule is not physically or chemically altered. Figure 3 shows how gel filtration can be used to separate ^125^I-protein (in this case, OVA) from free, unbound ^125^I. As ^125^I-radiolabelling of OVA does not alter the physicochemical properties of OVA, all the OVA added to the gel filtration column is eluted over a small volume range (~12 to 20 min; Figure 3) and this elution time/volume was not influenced by the presence of the ^125^I-label (Figure 3). Commonly the binding efficiency of ^125^I to protein is not 100% efficient and therefore due to its reduced size excess ‘free’ ^125^I elutes at a slower rate: with the outlined set-up free ^125^I eluted 26 to 40 min after addition to the column (Figure 3).

Using the above described method, a range of ^125^I-radiolabelled proteins can be produced and used to investigate protein loading within in range of systems including liposomes [6,15,23], niosomes [16] and microspheres [15,18]. Using this method we have also shown that the protein antigens Ag85B-ESAT-6, CtH1 and lysozyme can all adsorb to cationic liposomes to varying degrees [22,23] with the pI of the proteins playing a significant role in surface adsorption.

### 3.2. Radiolabelling of liposomes

**Figure 4:**
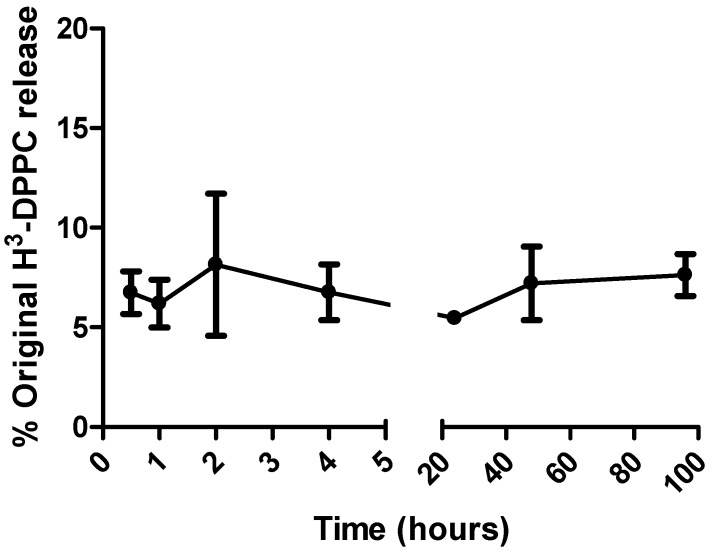
Release profile of ^3^H-DPPC from DDA:TDB liposomes when kept in simulated *in vivo* conditions (37 °C, 50% FCS). Results denote mean (*n = 3*) ± SD.

In order to verify that liposome membrane degradation with release of the radiolabel tracer did not occur upon exposure of liposomes to the *in vivo* environment, stability studies were conducted at 37 °C in a high protein environment. Dialysis was used to investigate the retention of the lipid label ^3^H-DPPC in liposomal bilayers. A dialysis membrane with a pore size large enough to allow large molecules to pass, yet retain liposomes, was chosen. Figure 4 shows the release profile of ^3^H-DPPC from cationic DDA:TDB liposomes; less than 10% of the ^3^H-DPPC radiolabel was detected in the dialysis buffer over the initial 96 h. The dialysis membrane had a molecular weight cut off (MWCO) of 12-14 kDa and whilst 3D measurements such as molecular size cannot be exactly correlated with 2D measurements such as length, the pore size was large enough to allow the movement of DPPC (MW 733) but retain liposomes which had a vesicle diameter of approximately 500 nm. As a rough guide, a MWCO of 12-14 kDa translates to a 2D measurement of approximately 2 nm. Consequently, liposomes of approximately 500 nm will be retained whilst allowing DPPC and small micellar systems to pass through. With regard to free DPPC and micelle formation, the quantities of ^3^H-DPPC outlined in section 2.4 result in a DPPC molarity of 0.42 nM (assuming there is 100% ^3^H-DPPC immediately). This is just below the cited CMC of DPPC 0.46 nM so therefore it is safe to assume that even if all the ^3^H-DPPC left the liposomes immediately, micelle formation would not occur.

### 3.3. Measuring dual-radiolabelled preparations in tissues

**Figure 5:**
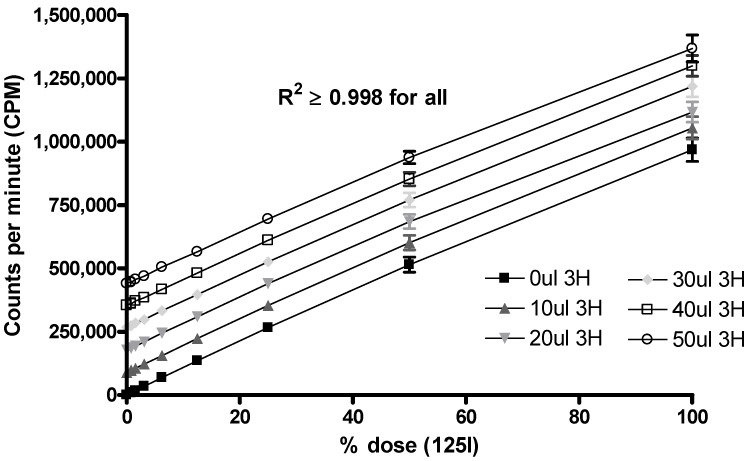
Graph showing how addition of ^3^H (2-25 kBq) adds linearly to ^125^I detection when measured using a scintillation counter. ^3^H was added to serially diluted ^125^I samples representing between 0.5–100% of the ^125^I dose. The R^2^ value for all data sets was equal to or more than 0.998. Results denote mean (*n = 3*) ± SD.

Herein we have described how ^3^H-radiolabelled liposomes and ^125^I-radiolabelled antigen can be used separately to study the stability of vaccines under conditions stimulating the *in vivo* environment. Whilst the application of dual-radiolabelled vaccines provides an ideal method to measure the pharmacokinetic properties and tissue distribution of both the liposome and antigen, the presence of two radioisotopes in the same tissue is problematic for accurate ^3^H quantification as ^125^I is also detected using scintillation counting (however ^3^H is not detected using γ-counting). To overcome this problem we devised a method whereby the ^125^I could be factored out by the use of a standard curve. Triplicate samples of ^125^I starting at an activity equivalent to the dose administered *in vivo* (~100 kBq) were diluted 2-fold until background levels (~20 cpms) were reached. These samples were counted using a γ-counter and then transferred and processed as the tissue samples would be (see section 2.5). The samples were then counted on a scintillation counter using the same ^3^H detection protocol as used for the tissue samples. A plot of the cpm values derived from the γ-counter (x-axis) against the cpm values derived from the scintillation counter (y-axis) was made and the line of best fit and equation derived for samples below 50,000 cpm (~2% of the dose or less) and those above 50,000 cpm (~2% of the dose or more). These two equations were used to calculate the effective interference that the ^125^I would have on the ^3^H values determined by scintillation counting. We found that the efficiency of ^125^I counting using our scintillation counters was approximately half of that seen by the γ-counter; however for each separate biodistribution study conducted a new ^125^I standard curve was produced as the machines counting efficacy can change and is also dependent on the amount of ^125^I in the sample. To investigate whether this relationship was linear, known volumes of ^3^H were added to the serially diluted ^125^I samples and counted on the scintillation counter. The results showed a linear relationship (Figure 5) indicating that there is no quenching effect between the two radioisotopes and that the above equation method to remove interfering ^125^I counts is valid.

### 3.4. Tracking the In Vivo Fate of Radiolabelled Systems

**Figure 6:**
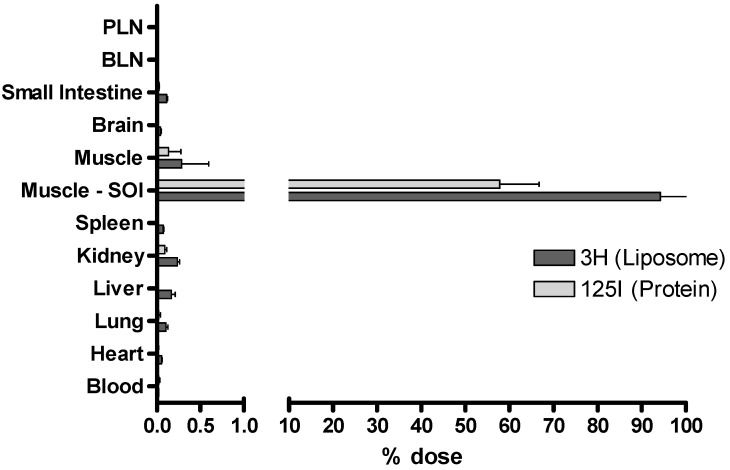
Dual-radiolabelling offers the possibility of detecting both ^3^H-labelled liposomes and ^125^I-labelled protein in a wide selection of tissues. A range of tissues were collected one day post injection and processed to determine the proportion of each radioisotope. Popliteal lymph node, PLN; brachial lymph node, BLN; site of injection, SOI. Results denote mean (*n = 3*) ± SD.

**Figure 7:**
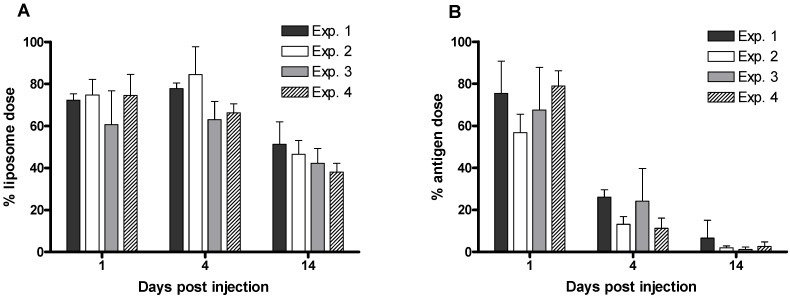
Reproducibility of dual-radiolabelling experiments involving ^3^H-radiolabelled liposomes (A) and ^125^I-radiolabelled protein (B). Four different experiments were conducted using the same liposome formulation as a control to measure the percentage of the injected dose still present at the injection site on days 1, 4 and 14 post injection. Results denote mean (*n = 3 or 5*) ± SD.

The method described for the processing of tissues and determination of the relevant quantities of ^3^H-radiolabelled liposomes and ^125^I-radiolabelled protein is suitable for a range of tissues. Figure 6 shows the proportions of each vaccine component detected in a selection of tissues. For the given example, the vaccine shows a characteristic depot-effect at the site of injection (SOI). However low levels (<0.01% of the injected dose) can also be detected in the lymph nodes. With respect to depot-forming vaccines, our studies have focussed on obtaining data from the site of injection as long term antigen retention at the SOI is one of the described mechanisms of adjuvant action [3]. We have conducted numerous studies whereby DDA:TDB liposomes adsorbing antigen were used as a control formulation. Figure 7 shows the strong reproducibility of these experiments and highlights the ability of this control liposome formulation to cause an antigen depot-effect at the SOI for a period of at least 14 days.

## 4. Conclusion

To conclude, we have shown that the use of two readily accessible radioisotopes, ^3^H and ^125^I, can be used simultaneously to detect vaccine components after injection. Using dialysis and centrifugation techniques we have shown that the radiolabel does not dissociate significantly from either the liposome or protein after exposure to an environment simulating *in vivo* conditions. Finally, this dual-radiolabelling is reproducible and applicable for vaccine studies for the detection of vaccines in a range of tissues.

## References

[1] Hevesy G.d., Paneth F. (1913). The solubility of lead sulphide and lead chromate. Z. Anorg. Chem..

[2] Hevesy G.d., Chievitz O. (1935). Radioactive indicators in the study of phosphorous in the metabolism in rats. Nature.

[3] Schijns V.E.J.C. (2000). Immunological concepts of vaccine adjuvant activity. Curr. Opin. Immunol..

[4] (2005). Guidelines on Adjuvants in Vaccines for Human Use.

[5] Davidsen J., Rosenkrands I., Christensen D., Vangala A., Kirby D., Perrie Y., Agger E.M., Andersen P. (2005). Characterisation of cationic liposomes based on dimethyldioctadecylammonium and synthetic cord factor from *M. tuberculosis* (trehalose 6,6'-dibehenate) - A novel adjuvant inducing both strong CMI and antibody responses. Biochim. Biophys. Acta..

[6] Vangala A., Bramwell V.W., McNeil S., Christensen D., Agger E.M., Perrie Y. (2007). Comparison of vesicle based antigen delivery systems for delivery of hepatitis B surface antigen. J. Control. Release.

[7] Perrie Y., Gregoriadis G. (2000). Liposome-entrapped plasmid DNA: characterisation studies. Biochim. Biophys. Acta..

[8] Bhowruth V., Minnikin D.E., Agger E.M., Andersen P., Bramwell V.W., Perrie Y., Besra G.S. (2009). Adjuvant Properties of a Simplified C_32_ Monomycolyl Glycerol Analogue. Bioorg. Med. Chem. Lett..

[9] Holmes K.L., Lantz L.M. (2001). Protein labeling with fluorescent probes. Methods Cell Biol..

[10] Medina L.A., Klipper R., Phillips W.T., Goins B. (2004). Pharmacokinetics and biodistribution of [^111^In]-avidin and [^99m^Tc]-biotin-liposomes injected in the pleural space for the targeting of mediastinal nodes. Nucl. Med. Biol..

[11] Oussoren C., Zuidema J., Crommelin D.J.A., Storm G. (1997). Lymphatic uptake and biodistribution of liposomes after subcutaneous injection. II. Influence of liposomal size, lipid composition and lipid dose. Biochimica et Biophysica Acta.

[12] Oussoren C., Velinova M., Scherphof G., Want J.J.v.d., Rooijen N.v., Storm G. (1998). Lymphatic uptake and biodistribution of liposomes after subcutaneous injection. IV. Fate of liposomes in reginal lymph nodes. Biochimica et Biophysica Acta.

[13] Niven R., Pearlman R., Wedeking T., Mackeigen J., Noker P., Simpson-Herren L., Smith J.G. (1998). Biodistribution of Radiolabeled Lipid-DNA Complexes and DNA in Mice. J. Pharm. Sci..

[14] Soundararajana A., Bao A., Phillips W.T., III R.P., Goins B.A. (2009). [^186^Re]Liposomal doxorubicin (Doxil): in vitro stability, pharmacokinetics, imaging and biodistribution in a head and neck squamous cell carcinoma xenograft model. Nucl. Med. Biol..

[15] Kirby D., Rosenkrands I., Agger E.M., Andersen P., Coombes A.G.A., Perrie Y. (2008). Liposomes act as stronger sub-unit vaccine adjuvants when compared to microspheres. J. Drug Target.

[16] Vangala A., Kirby D., Rosenkrands I., Agger E.M., Andersen P., Perrie Y. (2006). A Comparative study of cationic liposome and niosome-based systems for protein subunit vaccines: characterisation, environmental scanning electron microscopy and immunisation studies in mice. J. Pharm. Pharmacol..

[17] Perrie Y., Mohammed A.R., Vangala A., McNeil S. (2007). Environmental scanning electron microscopy offers real-time morphological analysis of liposomes and niosomes. J. Liposome Res..

[18] Kirby D., Rosenkrands I., Agger E.M., Andersen P., Coombes A.G.A., Perrie Y. (2008). PLGA microspheres for the delivery of a novel subunit TB vaccine. J Drug Target..

[19] Bangham A.D., Standish M.M., Watkins J.C. (1965). Diffusion of univalent ions across the lamellae of swollen phospholipids. J. Mol. Biol..

[20] Salacinski P.R.P., McLean C., Sykes J.E.C., Clement-Jones V.V., Lowry P.J. (1981). Iodination of proteins, glycoproteins, and peptides using a solid-phase oxidizing agent, 1,3,4,6-tetrachloro-3α,6α-diphenyl glycoluril (Iodogen). Anal. Biochem..

[21] Pause E., Bormer O., Nustad K.  (1982). Radioiodination of proteins with the IODO-GEN method. Radioimmunoassay and Related Procedures in Medicine.

[22] Henriksen-Lacey M., Christensen D., Bramwell V.W., Lindenstrøm T., Agger E.M., Andersen P., Perrie Y. (2010). Liposomal cationic charge and antigen adsorption are important properties for the efficient deposition of antigen at the injection site and immunogenicity of the vaccine. J. Control. Release.

[23] Henriksen-Lacey M., Bramwell V.W., Christensen D., Agger E.M., Andersen P., Perrie Y. (2009). Liposomes based on dimethyldioctadecylammonium promote a depot effect and enhance immunogenicity of soluble antigen. J. Control. Release.

